# Effect of Changing Electronic Health Record Opioid Analgesic Dispense Quantity Defaults on the Quantity Prescribed: A Cluster Randomized Clinical Trial

**DOI:** 10.1001/jamanetworkopen.2021.7481

**Published:** 2021-04-22

**Authors:** Marcus A. Bachhuber, Denis Nash, William N. Southern, Moonseong Heo, Matthew Berger, Mark Schepis, Manu Thakral, Chinazo O. Cunningham

**Affiliations:** 1 Division of General Internal Medicine, Department of Medicine, Montefiore Medical Center/Albert Einstein College of Medicine, Bronx, New York; 2 Now with Section of Community and Population Medicine, Louisiana State University Health Sciences Center–New Orleans; 3 Institute for Implementation Science in Population Health, City University of New York, New York; 4 Graduate School of Public Health and Health Policy, Department of Epidemiology and Biostatistics, City University of New York, New York, New York; 5 Division of Hospital Medicine, Montefiore Medical Center/Albert Einstein College of Medicine, Bronx, New York; 6 Now with College of Behavioral, Social and Health Sciences, Department of Public Health Sciences, Clemson University, Clemson, South Carolina; 7 Montefiore Information Technology, Montefiore Medical Center, Bronx, New York; 8 College of Nursing and Health Sciences, University of Massachusetts Boston, Boston

## Abstract

**Importance:**

Interventions to improve judicious prescribing of opioid analgesics for acute pain are needed owing to the risks of diversion, misuse, and overdose.

**Objective:**

To assess the effect of modifying opioid analgesic prescribing defaults in the electronic health record (EHR) on prescribing and health service use.

**Design, Setting, and Participants:**

A cluster randomized clinical trial with 2 parallel arms was conducted between June 13, 2016, and June 13, 2018, in a large urban health care system comprising 32 primary care and 4 emergency department (ED) sites in the Bronx, New York. Data were analyzed using a difference-in-differences method from 6 months before implementation through 18 months after implementation. Data were analyzed from January 2019 to February 2020.

**Interventions:**

A default dispense quantity for new opioid analgesic prescriptions of 10 tablets (intervention) vs no change (control) in the EHR.

**Main Outcomes and Measures:**

The primary outcome was the quantity of opioid analgesics prescribed with the new default prescription. Secondary outcomes were opioid analgesic reorders and health service use within 30 days after the new prescription. Intention-to-treat analysis was conducted.

**Results:**

Overall, 21 331 patients received a new opioid analgesic prescription from 490 prescribers. Comparing the intervention and control arms, site, prescriber, and patient characteristics were similar. For the new prescription, compared with the control arm, patients in the intervention arm had significantly more prescriptions for 10 tablets or fewer (7.6 percentage points; 95% CI, 6.1-9.2 percentage points), a lower number of tablets prescribed (−2.1 tablets; 95% CI, −3.3 to −0.9 tablets), and lower morphine milligram equivalents (MME) prescribed (−14.6 MME; 95% CI, −22.6 to −6.6 MME). Within 30 days after the new prescription, significant differences remained in the number of tablets prescribed (−2.7 tablets; 95% CI, −4.8 to −0.6 tablets), but not MME (−15.8 MME; 95% CI, −33.8 to 2.2 MME). Within this 30-day period, there were no significant differences between the arms in health service use.

**Conclusions and Relevance:**

In this study, implementation of a uniform reduced default dispense quantity of 10 tablets for opioid analgesic prescriptions led to a modest reduction in the quantity prescribed initially, without significantly increasing health service use. However, during 30 days after implementation, the influence on prescribing was mixed. Reducing EHR default dispense quantities for opioid analgesics is a feasible strategy that can be widely disseminated and may modestly reduce prescribing.

**Trial Registration:**

ClinicalTrials.gov Identifier: NCT03003832

## Key Points

**Question:**

Does reducing the default number of tablets for opioid analgesic prescriptions in the electronic health record influence prescribing or other outcomes?

**Findings:**

In this cluster randomized clinical trial of 32 primary care and 4 emergency department sites, with a total of 21 331 prescriptions, a reduced uniform default dispense quantity of 10 tablets led to an increase in the percentage of prescriptions for 10 tablets or fewer (7.6 percentage points). No significant difference in health service use was noted.

**Meaning:**

Findings of this study suggest that implementation of a reduced uniform opioid analgesic prescribing default is a feasible intervention that can modestly reduce prescribing.

## Teaser

This cluster randomized clinical trial examines the effect of reducing the default prescribing of opioid analgesics in the electronic health record (EHR) to 10 tablets in primary care and emergency department settings.

## Introduction

Opioid analgesics prescribed for acute noncancer pain are associated with risks such as diversion, misuse, and overdose.^1,2,3,4,5,6,7,8,9,10,11^ Efforts to promote judicious opioid prescribing for acute pain have included prescriber education, clinical guidelines, and legislative mandates. However, with widespread use of electronic health records (EHRs) and the demonstrated efficacy of EHR-based interventions in influencing care,^12,13,14^ technology offers new scalable opportunities, such as modifying opioid analgesic prescribing defaults.

Prescribing defaults refer to configuration settings within the EHR that prepopulate certain fields in new prescription orders, such as the number of tablets to dispense. Although fully modifiable by the prescriber, these prepopulated numbers can guide prescribers toward a lower quantity than they otherwise might have chosen. In 10 recent studies, implementing a new or lower default dispense quantity for opioid analgesics, either alone or as part of a package of interventions, was associated with reductions in the quantity of new prescriptions.^15,16,17,18,19,20,21,22,23,24^ In 2 studies, removing defaults, and therefore requiring prescribers to make an active choice with each prescription, yielded mixed results.^23,25^

Given evidence that many patients do not use the full amount of opioid analgesic tablets dispensed and retain the remainder,^5,26,27,28,29^ reducing the quantity prescribed may better approximate the amount used. However, a reduced default may result in unintended consequences. If the default dispense quantity is too low, for example, some patients may need additional medication or seek medical attention for uncontrolled pain. Furthermore, given that patterns of opioid analgesic prescribing are changing, the use of rigorous, controlled, study designs is necessary. Uncontrolled analyses (eg, pre-post analyses) may suggest an intervention is efficacious when changes in prescribing would have happened regardless of the intervention, for example, due to policy or regulatory changes.

The goal of this study was to assess the effect of a uniform reduced default dispense quantity for new opioid analgesic prescriptions on the quantity prescribed. We hypothesized that, compared with control, reducing the default dispense quantity would lead to a higher percentage of prescriptions written for the new reduced default number of tablets or fewer. In addition, compared with the control (ie, no intervention), we hypothesized that reducing the default dispense quantity would not lead to a significant increase in subsequent opioid analgesic prescription reorders or primary care visits, emergency department (ED) visits, or hospitalizations. For this study, we selected a cluster randomized design (ie, at the level of the clinical site) to mitigate the risk of contamination and take advantage of possible peer effects to increase the effectiveness of the intervention.

## Methods

### Study Design and Participants

Between June 13, 2016, and June 13, 2018, we conducted a cluster randomized clinical trial with 2 parallel arms among all primary care (n = 32) and ED (n = 4) sites within Montefiore Medical Center. Data were analyzed from January 2019 to February 2020. Montefiore Medical Center is the largest health care system in the Bronx (a borough of New York City) and provides comprehensive primary, specialty, surgical, and emergency care with more than 3 million patient visits annually. Montefiore Medical Center uses the Epic EHR system (Epic Systems Corp), which was the primary source of study data. This study followed the Consolidated Standards of Reporting Trials (CONSORT) reporting guideline extension for cluster randomized clinical trials.

To determine the effect of the intervention, we conducted an intention-to-treat analysis of outcomes for patients who (1) received a new opioid analgesic prescription at a study site, defined as no other opioid analgesic prescription of any type in the preceding 6 months (a definition used in previous cohort studies)^30,31^; (2) were aged 18 years or older; and (3) had no *International Statistical Classification of Diseases, 10th Revision, Clinical Modification* (*ICD-10-CM*) diagnosis code for cancer within 1 year before the new opioid analgesic prescription. For patients receiving more than 1 new opioid analgesic prescription during the study period, we only included the first prescription.

The protocol of this trial has been previously published^32^ and is included in its original form in Supplement 1. The protocol was approved, with a waiver of informed consent per the standard criteria as described in 45 CFR 46.116, by the Montefiore Medical Center/Albert Einstein College of Medicine Institutional Review Board. There were no changes to the methods after the trial was initiated.

### Intervention and Control Conditions

The intervention condition was a site-level change to the EHR to implement a uniform, reduced, default dispense quantity of 10 tablets for new opioid analgesic prescriptions. We chose this default dispense quantity because it represents a 3- to 5-day supply for most people, based on our clinical judgment and a review of available literature at the time.^32^ This default number of tablets prepopulated in all new opioid prescription orders in the intervention condition and remained fully modifiable. The intervention included all short-acting opioid analgesics commonly used to treat acute pain: immediate-release oxycodone, immediate-release hydrocodone, tramadol, and codeine. We included all brand and generic formulations and all tablet strengths and coformulations with acetaminophen.

The usual EHR served as the control condition. Depending on the exact medication product, the preexisting default number of tablets was typically 30 or blank, with several outliers.^32^ Of 44 products, the intervention reduced the default dispense quantity for most products (n = 36 for primary care, n = 37 for EDs) and created a default dispense quantity for several products (n = 8 for primary care, n = 7 for EDs).

To determine the effect of the intervention, we analyzed patient-level outcomes from 6 months before implementation (baseline period) to 18 months after implementation. Our primary outcome was the quantity of opioid analgesic prescribed in the new prescription, consisting of 3 measures: (1) 10 or fewer tablets (primary measure, dichotomous), (2) number of tablets prescribed (continuous), and (3) total morphine milligram equivalents (MME) prescribed (continuous). Secondary outcomes included opioid analgesic prescription reorders of either the same or a different opioid and health service use during the 30-day period after the new prescription. We measured reorders as (1) any reorder (dichotomous), (2) total number of tablets prescribed during the 30-day period after the new prescription and inclusive of the new prescription (continuous), and (3) total MME prescribed during the 30-day period after the new prescription and inclusive of the new prescription (continuous). We measured health service use as any (1) primary care visit, (2) ED visit, or (3) hospitalization (all dichotomous) during the 30-day period after the new prescription. There were no changes to trial outcomes after trial initiation.

### Participant Characteristics and Randomization

In addition to primary and secondary outcomes, we collected data on prescriber and patient characteristics that were potential confounders. For prescribers, we collected sex and years since graduation from medical school. For patients, we collected demographic information (age, sex, and race/ethnicity as recorded in the EHR). We also noted the pain diagnosis at the visit in which the new opioid analgesic was prescribed (ie, the indication for the opioid analgesic) in addition to the documented presence or absence of a history of psychiatric illness and substance use disorder within 1 year before the new prescription. We identified pain diagnoses, psychiatric illness, and substance use disorder through grouping *ICD-10-CM* diagnosis codes into clinically meaningful categories.^33,34,35^

The unit of randomization was the site (ie, cluster randomization) and we randomized in matched pairs. First, we stratified sites by type (ie, primary care vs ED). Next, within primary care sites, we stratified by specialty (ie, internal medicine and family medicine) and whether the setting is a training site for resident physicians. In addition, within strata, we used optimal nonbipartite matching to pair sites based on the number of new opioid analgesic prescriptions, number of visits, and percentage of patients with commercial insurance.^36^ Emergency department sites had large differences in visit volume and so we divided these sites into a pair consisting of the largest ED vs the 3 other smaller EDs combined. Randomization of sites was conducted by the study statistician (M.H.) and provided directly to the health information technology department. Other study investigators were blinded to randomization assignment.

### Statistical Analysis

First, we used descriptive statistics to compare site, prescriber, and patient characteristics between the intervention and control arms. Next, we analyzed the change in outcomes using a difference-in-differences (DID) analysis. One of the key assumptions of DID analysis is parallel trends before intervention. To test this assumption, we estimated the significance of an interaction term between study allocation (intervention/control) and time (month) in the preintervention period for all primary outcomes.

For the main DID analysis, we used generalized linear mixed regression models. For dichotomous outcomes, we used linear probability models. We chose this method to facilitate estimation given the hierarchical nature of the data and provide for easily interpretable results that would be useful for clinicians. For continuous outcomes, we used models with a γ distribution and a log link, selected based on the distributions of the outcome variables.^32^ To adjust for potential changes in composition over time, we included relevant site characteristics (number of new opioid analgesic prescriptions, the number of visits, and percentage of patients with commercial insurance), prescriber characteristics (sex and years since medical school graduation), and patient characteristics (age, sex, race/ethnicity, pain diagnosis, history of substance use disorder, and history of psychiatric disorder) as covariates (fixed effects) in all models. To account for the nesting of patients within prescribers and prescribers within sites, for dichotomous outcomes we included random intercepts both at the prescriber level and at the matched site pair level. For continuous outcomes, models with random intercepts both at the prescriber level and at the matched site pair level did not converge and so we used models with a random intercept at the prescriber level only. For all estimates, we calculated heteroscedasticity robust (empirical) SEs.^37,38^

In addition to the main analysis, we conducted several exploratory analyses. First, we analyzed the durability of the intervention after 6 months by comparing the outcomes after that time period (6-18 months post intervention) with outcomes 0 to 6 months post intervention. Next, we analyzed the impact of the intervention stratified by site type (ie, primary care vs ED), medication type (eg, schedule II vs schedules III and IV), and by whether the 10 tablets represented a new default vs a reduction in a preexisting default for opioid prescribing. We conducted these analyses using a triple differences method, which estimates the difference in the outcome from before to after intervention in the intervention arm minus the difference in the outcome from before to after intervention in the control arm in one subgroup minus the comparable difference in the comparison subgroup. We conducted all analyses with SAS, version 9.4 (SAS Institute Inc) and Stata, version 15.1 (StataCorp LLC). A 2-sided value of *P* ≤ .05 was considered significant.

From preliminary data analyses, we estimated that the planned study would be powered to detect a change in primary outcome in the intervention arm over and above any change in the control arm of 4.4 to 4.7 percentage points.^32^ We believed this to represent a clinically meaningful difference owing to the scalable nature of the intervention.

## Results

**Figure 1.**
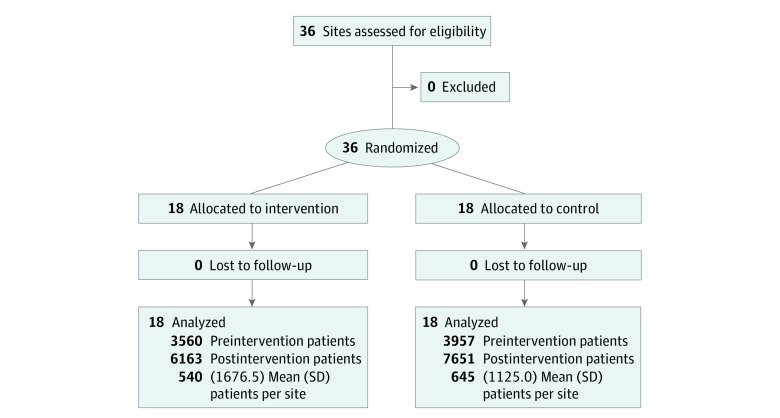
Consolidated Standards of Reporting Trials Flow Diagram

All primary care sites and ED sites were included, randomized, and analyzed with no losses to follow-up resulting in a total of 490 prescribers writing new opioid analgesic prescriptions for 21 331 patients (Figure 1). Compared with the control arm, sites in the intervention arm had a lower median number of visits (median, 8070; interquartile range [IQR], 3078-17 699 vs 9676; IQR, 4914-15 366) and new opioid analgesic prescriptions (median, 71; IQR, 17-103 vs 85; IQR, 49-182) before intervention as well as a lower percentage of patients with commercial insurance (median, 20.4; IQR, 18.0-29.1 vs 24.3; IQR, 19.8-35.4) (Table 1). For prescribers, characteristics between the intervention and control arm were similar. Of all prescribers, 26 (5.3%) prescribed an opioid analgesic at both an intervention and a control site after intervention, for a total of 981 prescriptions (7.1% of all analyzed prescriptions). In both the preintervention and postintervention periods, patients in the intervention and control arms had similar characteristics except, compared with the control arm, the intervention arm had a higher percentage of Hispanic/Latinx patients, a lower percentage of White patients, and a higher percentage of those with limb or extremity pain or arthritis in both the preintervention and postintervention periods (Table 2). We did not find any evidence to reject the parallel trends assumption.

**Table 1.** Characteristics of Clinical Sites and Prescribers

Characteristic	Intervention arm	Control arm
Sites		
No.	18	18
Primary care site, No. (%)	17 (94)	15 (83)
Training site for resident physicians, No. (%)	6 (33)	4 (22)
No. of visits before intervention, median (IQR)^a^	8070 (3078-17 699)	9676 (4914-15 366)
No. of new opioid analgesic prescriptions pre-intervention, median (IQR)^a^	71 (17-103)	85 (49-182)
Percentage commercial insurance, median (IQR)	20.4 (18.0-29.1)	24.3 (19.8-35.4)
Prescribers		
No.	232	258
Women, No. (%)	137 (59.1)	150 (58.1)
Years in practice, median (IQR)	10.9 (6.1-22.8)	11.0 (6.0-23.1)

Abbreviation: IQR, interquartile range.

^a^ Number in the 6-month preintervention period.

**Table 2.** Characteristics of Patients Before and After Intervention

Characteristic	No. (%)			
	Before intervention		After intervention	
	Intervention arm (n = 3560)	Control arm (n = 3957)	Intervention arm (n = 6163)	Control arm (n = 7651)
Age, median (IQR)	51.9 (38.2-63.1)	50.5 (37.4-62.5)	51.9 (37.5-63.2)	50.4 (36.2-62.2)
Women	2203 (61.9)	2571 (65.0)	3761 (61.0)	4848 (63.4)
Race/ethnicity				
Black, non-Hispanic	1090 (30.6)	1332 (33.7)	1819 (29.5)	2460 (32.2)
Hispanic/Latinx, any race	1678 (47.1)	1636 (41.3)	3044 (49.4)	3314 (43.3)
White, non-Hispanic/Latinx	192 (5.4)	439 (11.1)	344 (5.6)	806 (10.5)
Any other, multiple, or missing	600 (16.9)	550 (13.9)	956 (15.5)	1071 (14.0)
Pain diagnosis category				
Limb or extremity pain or arthritis	1222 (34.3)	1190 (30.1)	2205 (35.8)	2287 (29.9)
Abdominal, pelvic, or genitourinary	356 (10.0)	519 (13.1)	562 (9.1)	1054 (13.8)
Central or peripheral neuropathic pain	244 (6.9)	246 (6.2)	380 (6.2)	431 (5.6)
Back, neck, or craniofacial pain	679 (19.1)	743 (18.8)	1182 (19.2)	1297 (17.0)
Multiple pain diagnoses	478 (13.4)	609 (15.4)	848 (13.8)	1309 (17.1)
Other, unspecified, or missing diagnosis	581 (16.3)	650 (16.4)	986 (16.0)	1273 (16.6)
History of a mental health diagnosis	443 (12.4)	482 (12.2)	428 (6.9)	579 (7.6)
History of a substance use disorder diagnosis	52 (1.5)	64 (1.6)	59 (1.0)	93 (1.2)

Abbreviation: IQR, interquartile range.

**Figure 2.**
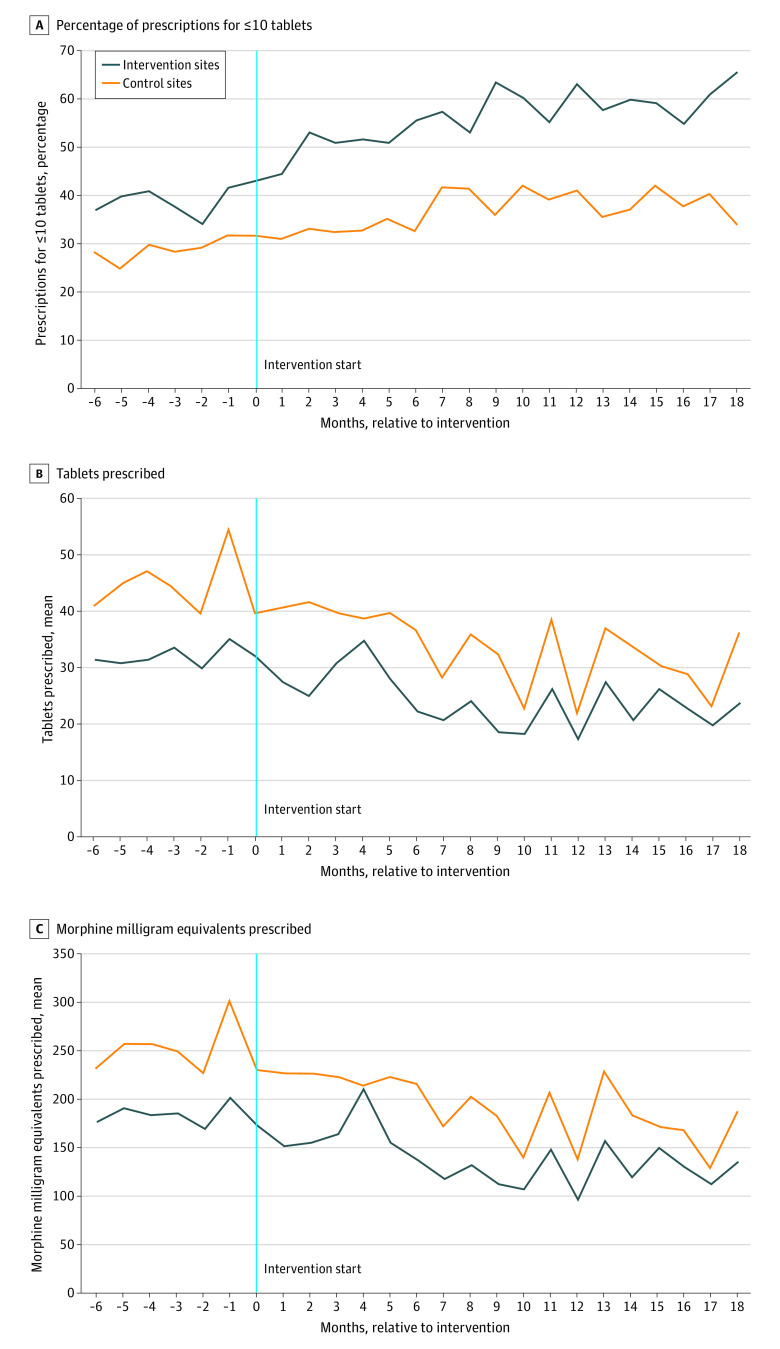
Unadjusted Primary Outcome Measures by Study Month A, Percentage of prescriptions for 10 or fewer tablets. B, Tablets prescribed. C, Morphine milligram equivalents prescribed.

Compared with the control arm, patients in the intervention arm had a significantly increased percentage of prescriptions for 10 tablets or fewer (adjusted DID, 7.6 percentage points; 95% CI, 6.1-9.2 tablets), lower number of tablets prescribed (adjusted DID, −2.1 tablets; 95% CI, −3.3 to −0.9 tablets), and lower total MME prescribed (adjusted DID, −14.6 MME; 95% CI, −22.6 to −6.6 MME) (Figure 2; Table 3). For secondary outcomes, during the 30-day period after the new prescription, compared with the control arm, patients in the intervention arm did not have a significant difference in opioid analgesic reorders (adjusted DID, 0.5 percentage points; 95% CI, −0.7 to 1.8), had a significantly lower number of tablets prescribed (adjusted DID, −2.7 tablets; 95% CI, −4.8 to −0.6), but showed no significant difference in the total MME prescribed (adjusted DID, −15.8 MME; 95% CI, −33.8 to 2.2). There were no significant differences between the arms in postprescription health service use. Comparing 6 to 18 months post intervention with 0 to 6 months post intervention between the intervention and control arms (ie, durability), the percentage of prescriptions for 10 tablets or fewer significantly increased in the intervention arm (adjusted DID, 2.0 percentage points; 95% CI, 0.9-3.1) with no significant differences in other outcomes (eTable 1 in Supplement 2).

**Table 3.** Primary and Secondary Outcomes

Outcome	Intervention arm (unadjusted)		Control arm (unadjusted)		Adjusted DID (95% CI)^a^	*P* value
	Pre (n = 3560)	Post (n = 6163)	Pre (n = 3957)	Post (n = 7651)		
**Primary**						
Dispense quantity ≤10 tablets, No. (%)	1364 (38.3)	3337 (54.1)	1122 (28.4)	2751 (36.0)	7.6 (6.1 to 9.2) percentage points	<.001
Tablets prescribed, No.						
Mean (SD)	32.2 (68.7)	25.3 (56.9)	45.1 (99.7)	34.7 (79.0)	−2.1 (−3.3 to −0.9)	<.001
Median (IQR)	15.0 (10.0-20.0)	10.0 (10.0-20.0)	15.0 (10.0-30.0)	12.0 (10.0-20.0)		
MME prescribed, No.						
Mean (SD)	184.9 (365.5)	144.9 (298.2)	253.8 (511.2)	197.8 (406.8)	−14.6 (−22.6 to −6.6)	<.001
Median (IQR)	90.0 (67.5-150.0)	75.0 (50.0-112.5)	90.0 (75.0-150.0)	90.0 (60.0-150.0)		
**Secondary**						
During the 30-d period after the index prescription						
Opioid analgesic prescription reorder, No. (%)	424 (11.9)	750 (12.2)	501 (12.7)	938 (12.3)	0.5 (−0.7 to 1.8) percentage points	.40
Total tablets prescribed, No.^b^						
Mean (SD)	37.8 (79.8)	30.8 (69.8)	52.1 (115.1)	40.9 (92.4)	−2.7 (−4.8 to −0.6)	.01
Median (IQR)	15.0 (10.0-30.0)	12.0 (10.0-24.0)	15.0 (12.0-40.0)	15.0 (10.0-30.0)		
Total MME prescribed, No.^b^						
Mean (SD)	229.6 (494.5)	186.4 (501.6)	304.9 (650.8)	244.7 (564.6)	−15.8 (−33.8 to 2.2)	.09
Median (IQR)	90.0 (75.0-180.0)	75.0 (52.5-150.0)	100.0 (75.0-225.0)	90.0 (67.5-172.5)		
Outpatient visit, No. (%)	228 (6.4)	398 (6.5)	263 (6.7)	554 (7.2)	−0.7 (−1.6 to 0.2) percentage points	.13
Emergency department visit, No. (%)	97 (2.7)	127 (2.1)	107 (2.7)	153 (2.0)	0.1 (−0.2 to 0.4) percentage points	.47
Hospitalization, No. (%)	36 (1.0)	67 (1.1)	54 (1.4)	98 (1.3)	0.2 (−0.08 to 0.4) percentage points	.18

Abbreviations: DID, difference in differences; IQR, interquartile range; MME, morphine milligram equivalent.

^a^ Difference in the outcome from the pre- to postintervention period in the intervention arm minus the difference in the outcome from the pre- to postintervention period in the control arm. All models were adjusted for site characteristics (number of visits, number of new opioid analgesic prescriptions, percentage of commercial insurance), prescriber characteristics (sex and years in practice), and patient characteristics (age, sex, race/ethnicity, pain diagnosis category, history of a mental health diagnosis, and history of a substance use disorder diagnosis).

^b^ Includes the index prescription and any opioid analgesic prescription reorders.

In exploratory analyses, there were no significant differences between primary care sites and ED sites for all outcomes (eTable 2 in Supplement 2). Among those prescribed schedule II opioid analgesics, compared with those receiving schedules III and IV opioid analgesics, the intervention increased prescription opioid analgesic reorders (adjusted triple difference, 4.8 percentage points; 95% CI, 2.4-7.1), total MME prescribed (adjusted triple difference, 38.6 MME; 95% CI, 4.2-73.1) and the percentage with an ED visit (adjusted triple difference, 1.7 percentage points; 95% CI, 1.1-2.3) (eTable 3 in Supplement 2). Results were similar comparing medications when the intervention represented a new default vs a reduction of an existing default (eTable 4 in Supplement 2).

## Discussion

In this cluster randomized clinical trial including 36 clinical sites, 490 prescribers, and 21 331 patients receiving a new opioid analgesic prescription, implementing a uniform default dispense quantity of 10 tablets led to a significant reduction in the quantity of opioids prescribed initially, without significantly increasing health services use. However, findings 30 days after the initial prescription were mixed. Together, our results indicate that reducing the default dispense quantity of opioid analgesics in an EHR modestly reduces opioid analgesic prescribing without increasing subsequent health services use.

The predominance of evidence indicates that defaults can influence opioid analgesic prescribing,^15,16,17,18,19,20,21,22,23,24,25^ and our study supports that conclusion. However, to date, few experimental studies have examined the association between prescribing defaults on subsequent prescriptions. In an observational study of postsurgical patients, a reduced default dispense quantity of 12 tablets was associated with a reduction in the quantity prescribed without a corresponding increase in prescription reorders.^16^ Although we had a similar finding, in one of our secondary outcomes, we also found no significant difference between the arms in the total MME prescribed during the 30-day period after the new prescription. Possible explanations for this finding include inadequate statistical power for this measure or that the reduced quantity of opioid analgesics received initially by intervention arm patients was offset by higher quantity prescription reorders later. For a more complete picture, our results suggest that future studies should include the total opioid analgesic prescriptions received over a follow-up period as an outcome.

Our findings also suggest that a default dispense quantity of 10 tablets may have unintended consequences for some patients. For those receiving schedule II opioids, we found that patients in the intervention arm were more likely than those in the control arm to receive a prescription reorder, receive prescriptions for a modestly higher quantity of opioids (ie, equivalent to approximately 5 oxycodone 5-mg tablets), and to visit the ED. For prescriptions, this result echoes a recent study that reported a default quantity of 10 tablets led to a higher mean quantity initially prescribed than a default quantity of 12 tablets.^19^ For ED visits, our finding was unanticipated and may be due to some patients seeking medical attention for undertreated pain. Overall, these results may be the result of, at least in part, the differences in pain condition and severity between patients receiving stronger vs weaker opioid analgesics. Further study is needed to identify optimal default quantities and our results additionally suggest that stratification by schedule is warranted.

### Limitations

This study has several limitations. First, although the trial was conducted at multiple sites, they were all within a single medical center and so the results may not be generalizable to other settings. In particular, the preexisting EHR configuration for opioid analgesic orders was set with a mixture of defaults and no defaults and so the control group may not reflect what a control group might be at another institution. Second, we were only able to obtain data from within our medical center and so outside prescriptions and visits were not captured, resulting in missingness of outcome data that may be nonrandom given imbalances in patient and site characteristics by study arm. Therefore, we may have underestimated the frequency of opioid analgesic reorders and health service use. This limitation may also bias the study findings if patients in one arm are more likely to obtain follow-up care within our medical center than patients in the other arm. Third, because our main data source was the EHR, we do not have information on whether prescriptions were dispensed or on patient-oriented outcomes, such as pain, functioning, or quality of life. Fourth, we used cluster randomization and a DID method with covariate adjustment to account for measured and unmeasured differences between the arms; however, there may be residual differences that bias our results. Fifth, a small number of prescribers (5.3%) wrote a prescription at both an intervention site and at a control site, resulting in contamination. However, this factor would be expected to bias our results toward a negative finding. Sixth, we selected a single, uniform, default dispense quantity of 10 tablets; a lower or higher default quantity or multiple default quantities depending on the medication order or setting, may have led to different results.

## Conclusions

In this randomized clinical trial, a uniform reduced default dispense quantity of 10 tablets for new opioid analgesic prescriptions decreased the quantity prescribed. Although the effects were modest, our findings support the efficacy of modifying EHR prescribing defaults in reducing opioid analgesic prescribing, which remains appealing owing to ease of implementation.

## Notes

Supplement 1. **Trial Protocol**

Supplement 2. **eTable 1.** Primary and Secondary Outcomes 6 to 18 Months Post Implementation Compared With 0 to 6 Months Post Implementation **eTable 2.** Primary and Secondary Outcomes in Primary Care Sites Compared With Emergency Department Sites **eTable 3.** Primary and Secondary Outcomes for Schedule II Medications Compared With Schedule III and IV Medications **eTable 4.** Primary and Secondary Outcomes When the Intervention Represented a New Default Compared With a Reduction in an Existing Default

Supplement 3. **Data Sharing Statement**
