# Supplementary material for: Effect of Changing Electronic Health Record Opioid Analgesic Dispense Quantity Defaults on the Quantity Prescribed: A Cluster Randomized Clinical Trial
Source: JAMA Netw Open. 2021 Apr 22;4(4):e217481. doi: 10.1001/jamanetworkopen.2021.7481 (PMC8063068; doi:10.1001/jamanetworkopen.2021.7481)
Supplement: Supplement 1. — Trial Protocol [file jamanetwopen-e217481-s001.pdf]

Trial Protocol:

Overview: This research centers on the implementation of default quantities for all new opioid analgesic prescriptions in the EHR. Within all Montefiore primary care and emergency department sites, we will conduct a cluster randomized controlled trial.

Study Participants: The primary participants are Montefiore providers. Eligible providers include those who provide primary care or emergency department care. We are seeking a waiver of informed consent for provider participants, similar to previous studies of EHR-based interventions delivered to providers.[1;2]

Intervention: The intervention consists of a change to the EHR interface so that new outpatient opioid analgesic prescriptions automatically default to a specified number of tablets (i.e., the "quantity dispensed" is pre-populated). This value is modifiable by providers who are free to change these values based on clinical factors. Re-orders of existing prescriptions (i.e., for people on chronic therapy) default to the original prescription, and will **not** be impacted by the intervention. The specific medications to be included are: tramadol, immediate-release oxycodone, immediate-release hydrocodone, and codeine. We will include all formulations (brand and generic) and dosage strengths of these medications, including co-formulations with acetaminophen or ibuprofen.

The default quantity will be 10 tablets. For opioid analgesic prescriptions, there are no specific studies addressing the optimal quantity that reduces the risks of harms while adequately treating pain. Generally, guidelines recommend a limited duration with early re-assessment.[3-5] The New York City Department of Health and Mental Hygiene currently recommends a 3-day supply for new opioid analgesic prescriptions to patients in acute care settings. Other states have adopted or are considering such a recommendation.[6]

All medications included in the intervention are short acting opioid analgesics and are typically written for 1 to 2 tablets every 4 to 6 hours, as needed. When calculating the number of days supplied, pharmacists use the maximum dose (i.e., 2 tablets every 4 hours). Therefore, the number of tablets needed for a 3-day supply, as calculated by a pharmacist, would be 36 tablets (2 tablets x 6 times per day x 3 days). However, patients often take fewer tablets per day since the instructions specify the medication is "as needed". In one study, after dermatological surgery, patients took a median of 3.7 opioid analgesic tablets total, about 1 pill per day.[7] In another study, after major open urologic surgery (i.e., open partial nephrectomy or radical cystectomy), patients took a median of 14 tablets total.[8] With a typical period of moderate-to-severe post-operative pain lasting about 5-10 days after hospital discharge, many patients therefore took about 2-3 tablets per day.

We chose a default of 10 tablets because we believe it represents at least a 3- to 5-day supply for most patients. With this change in defaults, providers are free to tailor the number of tablets dispensed based on clinical factors and judgment (i.e., the default is not hard-coded into the prescription).

Table 1. Data sources and data elements

Construct		Measure	Time period	Source
**Outcomes**				
Quantity of opioid analgesics prescribed †		At or below the default, number of tablets prescribed, number of MME*	Initial prescription	EHR
Opioid analgesic prescription re-orders		Number of tablets, number of MME	Within 30 days after initial prescription	EHR
Health service utilization		Primary care visit, ED visit, hospitalization	Within 30 days after initial prescription	EHR
**Covariates**				
Patient	Demographics	Age, gender, race/ethnicity	At initial prescription	EHR
	Insurance	Primary expected payer for visit	At initial prescription	EHR
	Pain diagnosis	Presence of extremity pain, back pain, neck pain, headache, fractures/contusions/injury, abdominal pain, headache, kidney or gallstones, other[11]	At initial prescription	EHR (ICD-10 codes)

	Comorbidities	History of substance use disorder, history of psychiatric illness	At initial prescription	EHR (ICD-10 codes)
Provider	Demographics	Age, gender, race/ethnicity	At initial prescription	MPD‡
	Specialty	Primary board certification	At initial prescription	MPD
	Years in practice	Years since graduation from medical school	At initial prescription	NYS§
Practice	Type	Acute or primary care	At initial prescription	EHR
	Teaching status	Site includes trainees (yes/no)	At initial prescription	MPD
*MME = morphine milligram equivalents †Primary outcome ‡MPD = Montefiore Provider Directory §Data from the NY State Office of the Professions				

<u>Control Condition.</u> The control condition is the usual EHR interface, termed standard of care (SOC).

<u>Data Sources:</u> To evaluate the impact of the intervention, we will analyze data collected in the course of routine clinical care. There will be three sources of data: 1) the Montefiore EHR 2) the Montefiore Provider Directory[9] and 3) medical license data from the New York State Office of the Professions.[10] Montefiore EHR data consist of prescription data, visit data, and patient data. The Montefiore Provider Directory and medical license data from the New York State Office of the Professions both contain demographic and training information about providers identified in EHR data.

<u>Data Elements:</u> We will be analyzing outcomes at the patient level. We will extract key variables from the following data sources (Table 1).

<u>Quantity of opioid analgesics prescribed.</u> For all opioid analgesic prescriptions from the Montefiore EHR, we will extract the medication name, dosage form/strength (i.e., number of milligrams per pill), formulation (i.e., immediate or extended-release), dosing instructions (i.e., number of tablets to take at one time and frequency of dosing), quantity dispensed, date written, and the prescribing provider. These data elements will be used to create the three measures of the primary outcome. First, we will classify all prescriptions as above, or at/below the default quantity. Second, we will extract the number of tablets to be dispensed. Finally, we will calculate the total morphine milligram equivalents (MME) to dispense. The use of MME standardizes comparisons between different types of opioid analgesics, as they have different strengths (i.e., milligrams per pill) and potencies. For example, if Provider A prescribes 15 tablets of oxycodone 10mg and Provider B prescribes 15 tablets of hydrocodone 5mg, both have prescribed the same number of tablets. However, Provider A prescribed a total of 150 mg of oxycodone and Provider B prescribed a total of 75 mg of hydrocodone. As oxycodone is about 1.5 times the potency of hydrocodone (i.e., 1 mg of oxycodone equals 1.5 MME and 1 mg of hydrocodone equals 1 MME),[12] Provider A prescribed 225 MME and Provider B prescribed 75 MME. Incorporating a measure of MME as an outcome is therefore vital in understanding the total amount of opioid prescribed.

<u>Opioid analgesic prescription re-orders.</u> Opioid analgesic prescription re-orders can occur if, for example, the patient does not receive an adequate supply of opioid analgesics in the initial prescription to treat his or her pain. From the Montefiore EHR, we will extract the same elements above for all other opioid analgesic prescriptions within 30 days after the initial prescription. For these prescriptions, we will record the number of tablets and the MMEs.

<u>Health service utilization.</u> Also from the Montefiore EHR, we will extract all outpatient and acute care visits, as well as any hospitalizations within 30 days after the initial opioid analgesic prescription.

<u>Patient characteristics (covariates):</u> We will obtain the primary diagnosis at the visit where the initial opioid analgesic was prescribed (i.e., the indication for the opioid analgesic) by grouping ICD-10 visit diagnosis codes into different clinically meaningful categories, for example: extremity pain, back pain, neck pain, headache, fractures/contusions/injury, abdominal pain, headache, kidney or gallstones, and other.[11] History of psychiatric illness and history of substance use disorder will be gathered from extracting all of the patients' ICD-10 diagnosis codes.

Data Collection: We will identify all patients age ≥ 18 years old who received a new opioid analgesic prescription, defined as one without another opioid analgesic prescription in the preceding 6 months, a definition used in previous cohort studies.[11;13] Patients will be excluded if they have a cancer diagnosis code within 1 year of the prescription. We will extract 6 months of data before intervention implementation and 18 months of data after implementation.
Data Management: After merging all data elements to create a final analytic dataset, we will strip all patient identifiers. Electronic data will be stored on a password-protected server, which will be backed up daily and can only be accessed by the research team.
Randomization: Study sites differ greatly in visit volume and characteristics; therefore, we will randomize in matched pairs to avoid a major imbalance which could threaten study validity. For randomization, we will stratify sites by type (i.e., primary care versus emergency department). Further, within primary care sites, prescribing patterns and the intervention's impact may differ by specialty (i.e., internal medicine and family medicine) and whether the site is a training site for resident physicians. Therefore, we will stratify on these variables as well. Within strata, we will use optimal non-bipartite matching to pair sites based on the number of new opioid analgesic prescriptions, the number of visits, and the percentage of patients with commercial insurance. For ED sites, given the very large differences in visit volume, we will divide the 4 sites into a "pair" consisting of the largest ED versus the 3 other smaller EDs combined.
Blinding: Randomization of sites within pairs will be conducted by the study statistician and provided directly to the health information technology department. Other study investigators will therefore be blind to randomization assignment.
Key Variables: Primary outcome: Quantity of opioid analgesics. We will use three measures of the primary outcome (described in detail above under "Opioid analgesic prescriptions"): 1) Whether or not the new opioid analgesic prescription was at, or below, the default quantity; 2) the number of tablets written; 3) the total MME written.

Secondary outcomes. In addition to the primary outcome, we will collect several measures of two secondary outcomes: *opioid analgesic prescription re-orders* and *health service utilization* (Table 1). For opioid analgesic prescription re-orders, we will analyze the number of tablets in subsequent prescriptions and the number of MME. For health service utilization, we will count the number of outpatient visits in addition to hospitalizations.

Other covariates. We will obtain other covariates as described in Table 1.

Analytic Plan:

Assessment of Randomization. To assess randomization, we will compare all provider and patient characteristics between the intervention and SOC arms (Table 1). Characteristics that are potential confounders will be included as covariates in regression models below.

Hypothesis 1: Compared to SOC, the intervention will lead to a higher percentage of prescriptions at, or below, the corresponding defaults. For this analysis, we will use a differences-in-differences (DID) approach to analyze the impact of the intervention. In a DID approach, the effectiveness of the intervention is determined by comparing the change in the intervention group's outcomes from pre-intervention to post-intervention to the change in the SOC group's outcomes from pre-intervention to post-intervention.[14;15] While delivery of the intervention is randomized at the practice site level, using data both pre- and post-intervention and a DID analysis has several advantages. First, DID will allow us to account for changes in prescribing due to factors other than the intervention (e.g., increased awareness of the problem of opioid analgesic misuse). Second, by using data from pre- and post-implementation of the intervention, the number of data points increases, and our ability to detect an impact from the intervention also increases. Third, while we can include covariates to adjust for randomization imbalance (see Assessment of Randomization above), DID will allow us to account for residual group-level heterogeneity such as differences in case mix or hard-to-measure factors like overall quality of care between intervention or SOC sites.[15] To assess the validity of DID assumptions, we will analyze pre-intervention data by testing for parallel trends.

To analyze the effect of intervention on the quantity of opioids prescribed, we will calculate the percentage of prescriptions in the intervention arm and SOC arm that are less than or equal to the default (the primary measure). We will do this for both pre-implementation and post-implementation. We will conduct the DID analysis using generalized linear mixed regression models (SAS PROC GLIMMIX). To account for clustering, we will include a random intercept at the provider level and at the matched site pair level. The model will be specified:

outcome = a + b1*_intervention_ + b2*(t) + b3*(_intervention_*t) + covariates + u

where **a** is the intercept, **t** is the study time (0 = pre-implementation and 1 = post-implementation), **intervention** indicates membership in the intervention arm (versus SOC), **covariates** are variables where imbalance was detected between the arms, **u** represents the provider-specific and site pair-specific random intercepts, and **b3**, the interaction term between intervention and study time, is the estimated effect of the intervention on the outcome.[15] Using a model with similar parameters, we will repeat this analysis for the secondary measures (number of tablets per prescription and MME per prescription).

Hypothesis 2: Compared to SOC, the intervention will not lead to a significant increase in opioid analgesic prescription re-orders or primary care visits, acute care visits, or hospitalizations. Using similar methods above, we will test the impact of the intervention on opioid analgesic prescription re-orders and health service utilization (secondary outcomes, Table 2). A separate regression model will be created for each outcome. For measures of opioid analgesic prescription re-orders (i.e., number of tablets and MME), we will use linear regression models. For measures of health service utilization, we will use similar models.

Power and Sample Size Analysis: From preliminary data analyses, we estimate eligible providers (N=~17 per site) will write a total of approximately 7,000 new opioid analgesic prescriptions (N=~11 prescriptions per provider) from the 36 sites during a six month post-intervention period. And, in the baseline period (i.e., 6 months prior), 32.7% of prescriptions will be for ≤ 10 tablets. From these parameters, we estimated the minimal detectable difference between study arms using a 3-level hierarchical model (i.e., patients clustered within providers who are clustered within matched site pairs). Because the intracluster correlation coefficient (ICC) is not known, we used a range of ICC from 0.01 to 0.1 at the patient level; only this level of ICC is needed for power analysis under our study design. Because any change in outcomes in the control arm is also unknown, we used a range of increases in the percentage of prescriptions for ≤ 10 tablets in the control arm of between 0 and 10 percentage points. Within this range of ICC, change in control arm outcomes, alpha=0.05, and power ≥ 80%, this study will be powered to detect a change in the intervention arm, over and above any change in the control arm, of 4.4 to 4.7 percentage points.

References

1. Linder JA, Rigotti NA, Schneider LI, Kelley JH, Brawarsky P, Haas JS. An electronic health record-based intervention to improve tobacco treatment in primary care: a cluster-randomized controlled trial. *Arch Intern Med* 2009;169:781-787.

2. Sequist TD, Gandhi TK, Karson AS et al. A randomized trial of electronic clinical reminders to improve quality of care for diabetes and coronary artery disease. *J Am Med Inform Assoc* 2005;12:431-437.

3. Cantrill SV, Brown MD, Carlisle RJ et al. Clinical policy: critical issues in the prescribing of opioids for adult patients in the emergency department. *Ann Emerg Med* 2012;60:499-525.

4. Epstein H, Hansen C, Thorson D. A protocol for addressing acute pain and prescribing opioids. *Minn Med* 2014;97:47-51.

5. Chou R, Qaseem A, Snow V et al. Diagnosis and treatment of low back pain: a joint clinical practice guideline from the American College of Physicians and the American Pain Society. *Ann Intern Med* 2007;147:478-491.

6. Ohio Department of Health. Ohio Emergency and Acute Care Facility Opioids and Other Controlled Substances (OOCS) Prescribing Guidelines. Published April 12,2012.

7. Harris K, Curtis J, Larsen B et al. Opioid pain medication use after dermatologic surgery: a prospective observational study of 212 dermatologic surgery patients. *JAMA Dermatol* 2013;149:317-321.

8. Bates C, Laciak R, Southwick A, Bishoff J. Overprescription of postoperative narcotics: a look at postoperative pain medication delivery, consumption and disposal in urological practice. *J Urol* 2011;185:551-555.

9. Montefiore Provider Directory. http://www.montefiore.org/physician-directory-1743

10. New York State Office of the Professions Physician Lookup. http://www.op.nysed.gov/opsearches.htm

11. Von KM, Saunders K, Thomas RG et al. De facto long-term opioid therapy for noncancer pain. *Clin J Pain* 2008;24:521-527.

12. Paulozzi LJ, Strickler GK, Kreiner PW, Koris CM. Controlled Substance Prescribing Patterns - Prescription Behavior Surveillance System, Eight States, 2013. *MMWR Surveill Summ* 2015;64:1-14.

13. Hooten WM, St Sauver JL, McGree ME, Jacobson DJ, Warner DO. Incidence and Risk Factors for Progression From Short-term to Episodic or Long-term Opioid Prescribing: A Population-Based Study. *Mayo Clin Proc* 2015;90:850-856.

14. Meyer BD. Natural and Quasi-Experiments in Economics. *Journal of Business & Economic Statistics* 1995;13:151-161.

15. Angrist J, Pischke J. *Mostly Harmless Econometrics: An Empiricist's Companion*. Princeton, NJ: Princeton University Press, 2009.

16. Vittinghoff E, Glidden DV, Shiboski SC, McCulloch CE. *Regression Methods in Biostatistics*. New York, NY: Springer, 2012.
