# Supplementary material for: Effect of Changing Electronic Health Record Opioid Analgesic Dispense Quantity Defaults on the Quantity Prescribed: A Cluster Randomized Clinical Trial
Source: JAMA Netw Open. 2021 Apr 22;4(4):e217481. doi: 10.1001/jamanetworkopen.2021.7481 (PMC8063068; doi:10.1001/jamanetworkopen.2021.7481)
Supplement: Supplement 2. — eTable 1. Primary and Secondary Outcomes 6 to 18 Months Post Implementation Compared With 0 to 6 Months Post Implementation eTable 2. Primary and Secondary Outcomes in Primary Care Sites Compared With Emergency Department Sites eTable 3. Primary and Secondary Outcomes for Schedule II Medications Compared With Schedule III and IV Medications eTable 4. Primary and Secondary Outcomes When the Intervention Represented a New Default Compared With a Reduction in an Existing Default [file jamanetwopen-e217481-s002.pdf]

Supplementary Online Content

Bachhuber MA, Nash D, Southern WN, et al. Effect of changing electronic health record opioid analgesic dispense quantity defaults on the quantity prescribed: a cluster randomized clinical trial. *JAMA Netw Open*. 2021;4(4):e217481. doi:10.1001/jamanetworkopen.2021.7481

e.Table 1. Primary and Secondary Outcomes 6 to 18 Months Post Implementation Compared With 0 To 6 Months Post Implementation

eTable 2. Primary and Secondary Outcomes in Primary Care Sites Compared With Emergency Department Sites

eTable 3. Primary and Secondary Outcomes for Schedule II Medications Compared With Schedule III and IV Medications

eTable 4. Primary and Secondary Outcomes When the Intervention Represented a New Default Compared With a Reduction in an Existing Default

This supplementary material has been provided by the authors to give readers additional information about their work.

© 2021 Bachhuber MA et al. *JAMA Network Open*

eTable 1. Primary and secondary outcomes 6 to 18 months post implementation compared with 0 to 6 months post implementation			
	Versus baseline		
	Adjusted difference-in-differences[a]	95% CI	P
Primary outcome			
Dispense quantity ≤ 10 tablets, %	2.0 percentage points	0.9 to 3.1	<0.001
Tablets prescribed, n	0.6 tablets	-0.7 to 1.8	0.37
Morphine milligram equivalents prescribed, n	2.6 morphine milligram equivalents	-6.8 to 11.9	0.59
Secondary outcomes			
Opioid analgesic prescription re-order during the 30-day period after the index prescription, %	1.0 percentage points	-0.3 to 2.2	0.13
Total tablets prescribed during the 30-day period after the index prescription, n[b]	2.2 tablets	-0.4 to 4.8	0.09
Total morphine milligram equivalents prescribed during the 30-day period after the index prescription, n[b]	3.7 morphine milligram equivalents	-24.8 to 32.3	0.78
Outpatient visit during the 30-day period after the index prescription, %	0.8 percentage points	-0.4 to 1.9	0.20
Emergency department visit during the 30-day period after the index prescription, %	0.005 percentage points	-0.2 to 0.3	0.97
Hospitalization during the 30-day period after the index prescription, %	0.1 percentage points	-0.2 to 0.4	0.39

[a]Refers to the difference in the outcome from pre- to post-intervention in the intervention arm minus the difference in the outcome from pre- to post-intervention in the control arm. All models adjust for site characteristics (number of visits, number of new opioid analgesic prescriptions, percentage commercial insurance), provider characteristics (gender and years in practice), and patient characteristics (age, gender, race/ethnicity, pain diagnosis category, history of a mental health diagnosis, and history of a substance use disorder diagnosis)
[b]Includes the index prescription and any opioid analgesic prescription reorders

© 2021 Bachhuber MA et al. *JAMA Network Open*

eTable 2. Primary and secondary outcomes in primary care sites compared with emergency department sites

	Primary care difference-in-differences, adjusted	95% CI	P	Emergency department difference-in-differences, adjusted	95% CI	P	Adjusted triple difference[a]	95% CI	P
Primary outcome									
Dispense quantity ≤ 10 tablets, %	5.8 percentage points	0.8 to 10.9	0.02	7.78 percentage points	7.72 to 7.84	<0.001	-1.9 percentage points	-7.0 to 3.1	0.45
Tablets prescribed, n	-4.3 tablets	-7.0 to 1.6	0.002	-1.4 tablets	-2.5 to -0.2	0.02	-2.9 tablets	-5.8 to 0.02	0.052
Morphine milligram equivalents prescribed, n	-27.2 morphine milligram equivalents	-45.9 to -8.5	0.004	-10.2 morphine milligram equivalents	-18.1 to -2.3	0.01	-17.0 morphine milligram equivalents	-37.2 to 3.2	0.10
Secondary outcomes									
Opioid analgesic prescription re-order during the 30-day period after the index prescription, %	-0.7 percentage points	-4.2 to 2.7	0.67	1.16 percentage points	1.08 to 1.24	<0.001	-1.9 percentage points	-5.4 to 1.6	0.28
Total tablets prescribed during the 30-day period after the index prescription, n[b]	-4.4 tablets	-8.0 to -0.7	0.02	-2.1 tablets	-4.6 to 0.4	0.09	-2.2 tablets	-6.6 to 2.2	0.32
Total morphine milligram equivalents prescribed during the 30-day period after the index prescription, n[b]	-28.7 morphine milligram equivalents	-58.5 to 1.1	0.06	-11.4 morphine milligram equivalents	-32.6 to 9.7	0.29	-17.3 morphine milligram equivalents	-53.5 to 18.9	0.35
Outpatient visit during the 30-day period after the index prescription, %	-0.6 percentage points	-3.6 to 2.4	0.69	-0.64 percentage points	-0.71 to -0.58	<0.001	0.03 percentage points	-3.0 to 3.1	0.99
Emergency department visit during the 30-day period after the index prescription, %	-0.1 percentage points	-0.8 to 0.5	0.73	0.29 percentage points	0.28 to 0.31	<0.001	-0.4 percentage points	-1.1 to 0.3	0.23
Hospitalization during the 30-day period after the index prescription, %	-0.1 percentage points	-0.7 to 0.5	0.76	0.3 percentage points	0.28 to 0.31	<0.001	-0.4 percentage points	-1.0 to 0.2	0.23

[a]Refers to the difference in the outcome from pre- to post-intervention in the intervention arm minus the difference in the outcome from pre- to post-intervention in the control arm in primary care sites minus the comparable difference in emergency department sites. All models adjust for site characteristics (number of visits, number of

© 2021 Bachhuber MA et al. *JAMA Network Open*

new opioid analgesic prescriptions, percentage commercial insurance), provider characteristics (gender and years in practice), and patient characteristics (age, gender, race/ethnicity, pain diagnosis category, history of a mental health diagnosis, and history of a substance use disorder diagnosis)

[b]Includes the index prescription and any opioid analgesic prescription reorders

© 2021 Bachhuber MA et al. *JAMA Network Open*

eTable 3. Primary and secondary outcomes for schedule II medications compared with schedule III and IV medications									
	Schedule II difference-in-differences, adjusted	95% CI	P	Schedule III/IV difference-in-differences, adjusted	95% CI	P	Adjusted triple difference[a]	95% CI	P
Primary outcome									
Dispense quantity ≤ 10 tablets, %	5.9 percentage points	4.9 to 6.9	<0.001	11.3 percentage points	5.9 to 16.8	<0.001	-5.4 percentage points	-10.9 to 0.005	0.050
Tablets prescribed, n	-1.8 tablets	-3.2 to -0.4	0.01	-2.9 tablets	-5.0 to -0.9	0.005	1.2 tablets	-1.4 to 3.7	0.37
Morphine milligram equivalents prescribed, n	-11.1 morphine milligram equivalents	-21.5 to -0.6	0.04	-16.6 morphine milligram equivalents	-29.5 to -3.6	0.01	5.5 morphine milligram equivalents	-11.6 to 22.7	0.53
Secondary outcomes									
Opioid analgesic prescription re-order during the 30-day period after the index prescription, %	2.9 percentage points	2.0 to 3.7	<0.001	-1.9 percentage points	-4.0 to 0.2	0.08	4.8 percentage points	2.4 to 7.1	<0.001
Total tablets prescribed during the 30-day period after the index prescription, n[b]	-1.1 tablets	-4.0 to 1.8	0.46	-4.9 tablets	-7.9 to -1.9	0.001	3.8 tablets	-0.3 to 7.9	0.07
Total morphine milligram equivalents prescribed during the 30-day period after the index prescription, n[b]	1.3 morphine milligram equivalents	-23.7 to 26.3	0.92	-37.3 morphine milligram equivalents	-59.5 to -15.2	0.001	38.6 morphine milligram equivalents	4.2 to 73.1	0.03
Outpatient visit during the 30-day period after the index prescription, %	-0.2 percentage points	-0.9 to 0.5	0.63	-1.0 percentage points	-3.0 to 1.0	0.31	0.9 percentage points	-1.3 to 3.0	0.44
Emergency department visit during the 30-day period after the index prescription, %	0.8 percentage points	0.6 to 1.1	<0.001	-0.8 percentage points	-1.3 to -0.4	<0.001	1.7 percentage points	1.1 to 2.3	<0.001
Hospitalization during the 30-day period after the index prescription, %	0.3 percentage points	0.1 to 0.4	0.002	0.07 percentage points	-0.5 to 0.6	0.82	0.2 percentage points	-0.4 to 0.8	0.53

[a]Refers to the difference in the outcome from pre- to post-intervention in the intervention arm minus the difference in the outcome from pre- to post-intervention in the control arm for schedule II prescriptions minus the comparable difference in schedule III and IV prescriptions. All models adjust for site characteristics (number of visits, number of new opioid analgesic prescriptions, percentage commercial insurance), provider characteristics (gender and years in practice), and patient characteristics (age, gender, race/ethnicity, pain diagnosis category, history of a mental health diagnosis, and history of a substance use disorder diagnosis)
[b]Includes the index prescription and any opioid analgesic prescription reorders

© 2021 Bachhuber MA et al. *JAMA Network Open*

eTable 4. Primary and secondary outcomes when the intervention represented a new default compared with a reduction in an existing default									
	New default difference-in-differences, adjusted	95% CI	P	Reduced existing default difference-in-differences, adjusted	95% CI	P	Adjusted triple difference[a]	95% CI	P
Primary outcome									
Dispense quantity ≤ 10 tablets, %	6.8 percentage points	5.1 to 8.5	<0.001	11.7 percentage points	5.8 to 1.8	<0.001	-4.9 percentage points	-11.4 to 1.5	0.13
Tablets prescribed, n	-2.0 tablets	-3.4 to -0.7	0.003	-2.9 tablets	-5.3 to -0.5	0.02	0.9 tablets	-1.8 to 3.6	0.53
Morphine milligram equivalents prescribed, n	-15.6 morphine milligram equivalents	-24.2 to -7.1	<0.001	-10.3 morphine milligram equivalents	-38.4 to 17.7	0.47	-5.3 morphine milligram equivalents	-34.8 to 24.1	0.72
Secondary outcomes									
Opioid analgesic prescription re-order during the 30-day period after the index prescription, %	0.7 percentage points	-0.5 to 1.9	0.24	-0.4 percentage points	-2.3 to 1.4	0.66	1.1 percentage points	-0.5 to 2.7	0.18
Total tablets prescribed during the 30-day period after the index prescription, n[b]	-2.5 tablets	-5.0 to -0.1	0.04	-4.2 tablets	-8.6 to 0.2	0.06	1.6 tablets	-3.4 to 6.7	0.53
Total morphine milligram equivalents prescribed during the 30-day period after the index prescription, n[b]	-12.3 morphine milligram equivalents	-33.6 to 8.9	0.26	-39.6 morphine milligram equivalents	-94.2 to 15.0	0.16	27.3 morphine milligram equivalents	-34.3 to 88.8	0.39
Outpatient visit during the 30-day period after the index prescription, %	-0.6 percentage points	-1.4 to 0.2	0.14	-0.9 percentage points	-3.0 to 1.1	0.36	0.3 percentage points	-1.6 to 2.3	0.74
Emergency department visit during the 30-day period after the index prescription, %	0.1 percentage points	-0.2 to 0.4	0.53	0.1 percentage points	-0.6 to 0.9	0.72	-0.04 percentage points	-0.8 to 0.7	0.91
Hospitalization during the 30-day period after the index prescription, %	0.09 percentage points	-0.1 to 0.3	0.38	0.6 percentage points	-1.0 to 2.2	0.48	-0.5 percentage points	-2.2 to 1.2	0.56

[a]Refers to the difference in the outcome from pre- to post-intervention in the intervention arm minus the difference in the outcome from pre- to post-intervention in the control arm in medication orders where the default was new minus the comparable difference in medication orders where the default represented a reduction of an existing default. All models adjust for site characteristics (number of visits, number of new opioid analgesic prescriptions, percentage commercial insurance), provider characteristics (gender and years in practice), and patient characteristics (age, gender, race/ethnicity, pain diagnosis category, history of a mental health diagnosis, and history of a substance use disorder diagnosis)
[b]Includes the index prescription and any opioid analgesic prescription reorders

© 2021 Bachhuber MA et al. *JAMA Network Open*
