# Supplementary material for: Effect of Changing Electronic Health Record Opioid Analgesic Dispense Quantity Defaults on the Quantity Prescribed: A Cluster Randomized Clinical Trial
Source: JAMA Netw Open. 2021 Apr 22;4(4):e217481. doi: 10.1001/jamanetworkopen.2021.7481 (PMC8063068; doi:10.1001/jamanetworkopen.2021.7481)
Supplement: Supplement 3. — Data Sharing Statement [file jamanetwopen-e217481-s003.pdf]

Data Sharing Statement

Bachhuber. Effect of Changing Electronic Health Record Opioid Analgesic Dispense Quantity Defaults on the Quantity Prescribed. *JAMA Netw Open*. Published April 22, 2021. doi:10.1001/jamanetworkopen.2021.7481

Data
Data available: No
